# Reliability of Artificial Intelligence Chatbots in Answering Patient-Oriented Questions About Endodontic Apical Lesions

**DOI:** 10.7759/cureus.107783

**Published:** 2026-04-27

**Authors:** Faraj Alotaiby, Waleed Almutairi

**Affiliations:** 1 Department of Oral and Maxillofacial Diagnostic Sciences, College of Dentistry, Qassim University, Buraydah, SAU; 2 Department of Conservative Dental Sciences, College of Dentistry, Qassim University, Buraydah, SAU

**Keywords:** artificial intelligence, chatgpt, deepseek, grok, periapical radiograph

## Abstract

Objective: This study aimed to evaluate the reliability and clinical appropriateness of responses generated by publicly accessible AI-based chatbot platforms (ChatGPT (OpenAI, San Francisco, CA, USA); Grok (xAI, Palo Alto, CA, USA); and DeepSeek (High-Flyer, Hangzhou, ZJ, CHN)) when addressing patient-oriented questions related to endodontic apical lesions.

Methods: A total of 15 standardized, non-technical questions were developed to simulate typical patient inquiries. The questions covered four domains: identification of apical lesions, differentiation between odontogenic and non-odontogenic causes, management and recommended next steps, and potential risks and follow-up considerations. Each question was independently submitted once to ChatGPT, Grok, and DeepSeek. Two expert evaluators (a board-certified endodontist and a board-certified oral and maxillofacial pathologist) assessed the responses using a five-point Likert scale based on reliability, clarity, and clinical appropriateness for patient education. Inter-rater agreement was assessed using Cohen’s kappa coefficient. Descriptive statistics were used to summarize response ratings, and differences among platforms were analyzed using the Kruskal-Wallis H test.

Results: All three AI platforms demonstrated high levels of expert agreement, with a Cohen’s kappa value of 0.85 indicating almost perfect inter-rater reliability. ChatGPT achieved the highest proportion of strongly agreeable responses, followed by Grok and DeepSeek. No statistically significant differences were observed among the platforms in agreement distributions (p = 0.36). Based on expert evaluation, ChatGPT responses tended to provide more detailed clinical explanations, whereas Grok and DeepSeek responses were perceived to use simpler and more accessible language.

Conclusion: Publicly accessible AI chatbots can provide generally reliable and clinically appropriate responses to patient-oriented questions concerning endodontic apical lesions. ChatGPT demonstrated higher informational accuracy, whereas Grok and DeepSeek offered clearer patient-centered communication. These findings support the cautious integration of AI chatbots as adjunct tools for patient education, while emphasizing the continued necessity of professional clinical judgment.

## Introduction

The rise of large language models (LLMs) has transformed how the public accesses medical and dental information. Designed to mimic human cognitive processes, AI has steadily found a place in healthcare, offering tools to assist in diagnostics, treatment planning, and patient education [1-3]. In dentistry, and particularly in endodontics, AI applications have shown promise in areas such as radiographic interpretation, the detection of periapical lesions, and clinical decision-making [2-5]. However, significant concerns have emerged regarding the reliability and clinical relevance of AI-generated information, particularly when utilized by individuals without professional training [4,5]. Although AI chatbots are capable of producing detailed and persuasive answers, they are not immune to errors, oversimplifications, and occasional misinformation [4,5]. This issue is particularly critical in endodontics, where diagnosing radiographic findings such as apical lesions demands refined clinical expertise and judgment [2,4].

As digital platforms become more accessible, patients increasingly turn to AI tools for advice on their oral health, often submitting dental radiographs and requesting interpretations or treatment recommendations. Many of these inquiries involve differentiating between odontogenic and non-odontogenic pathoses, an area that often requires nuanced clinical interpretation. While AI technologies offer a pathway to increasing public access to information, their reliability in accurately distinguishing among various types of oral pathologies remains uncertain.

This study aimed to evaluate the reliability and clinical appropriateness of information provided by publicly accessible AI-based chatbot platforms (ChatGPT (OpenAI, San Francisco, CA, USA); Grok (xAI, Palo Alto, CA, USA); and DeepSeek (High-Flyer, Hangzhou, ZJ, CHN)) in response to patient-style questions related to endodontic apical lesions. By applying an expert agreement-rating framework, the study assessed the suitability of AI-generated responses for patient-oriented communication rather than definitive diagnostic accuracy.

## Materials and methods

Prior to study initiation, the study protocol was reviewed and approved by the Institutional Review Board (IRB) of the College of Dentistry, Qassim University (Buraydah, Al-Qassim, SAU). The requirement for informed consent was waived by the IRB, as the study did not involve human participants, patient recruitment, or identifiable personal or clinical data. A total of 15 standardized questions were developed to simulate realistic inquiries commonly posed by dental patients after being informed of a radiographic finding near the root apex. The questions were formulated using non-technical, patient-oriented language and covered four domains: (i) identification of apical lesions, (ii) differentiation between odontogenic and non-odontogenic causes, (iii) management and recommended next steps, and (iv) potential risks and follow-up considerations (Table 1). These domains were internally defined for classification purposes; however, all questions were phrased using simplified, non-technical language to reflect how patients typically describe their concerns. The questions were designed to reflect typical concerns encountered in clinical practice rather than professional diagnostic scenarios.

**Table 1:** Questions simulating typical patient inquiries

Category	Question
Identifying the lesion	1. I have a dark spot at the end of my tooth on the X-ray. Is it dangerous?
	2. What could cause a shadow near the root of my tooth?
	3. Does a dark spot on a dental X-ray always mean I have an infection?
	4. Can you tell if the shadow on my X-ray is coming from the tooth or something else?
	5. How can I know if the lesion at the end of my tooth needs treatment?
Odontogenic vs. Non-odontogenic	6. Is the lesion on my X-ray coming from my tooth or from somewhere else?
	7. Can sinus problems cause shadows near teeth on X-rays?
	8. Could a cyst or tumor show up like a dental infection on an X-ray?
	9. If I don’t have any tooth pain, could the dark spot still be from a tooth problem?
Management and next steps	10. What should I do if I have a lesion near my tooth root?
	11. Do I need a root canal if there is a dark spot near the root?
	12. Could this kind of lesion go away on its own without treatment?
	13. Should I see a specialist if my X-ray shows a lesion?
Risk and follow-up	14. Can ignoring a lesion on the X-ray make it worse?
	15. How often do dentists check lesions like this to make sure they are not getting bigger?

Three publicly accessible LLM-based chatbot platforms were evaluated in this study: ChatGPT's GPT-4o version, Grok's Grok-1 version, and DeepSeek's DeepSeek-V2. These platforms were selected due to their widespread public availability and increasing use by individuals seeking health-related information online. Each platform was accessed using its standard public interface. The same set of predefined patient-style questions was entered independently into each chatbot in separate sessions, without follow-up prompts, clarifications, or iterative refinements. Responses were recorded verbatim at the time of generation to minimize variability related to session memory or contextual carryover.

Two independent evaluators, namely a board-certified endodontist and a board-certified oral and maxillofacial pathologist, assessed each response. Evaluations focused on the reliability and clinical appropriateness of the information provided from a patient-education perspective rather than definitive diagnosis. Each response was rated using a five-point Likert scale adapted from the methodology of the accepted manuscript, where 1 = strongly disagree, 2 = disagree, 3 = neutral, 4 = agree, and 5 = strongly agree.

Ratings reflected the degree to which each response was considered acceptable, clear, and appropriate for patient-oriented communication. Scores of 4 or 5 were considered indicative of reliable and clinically acceptable patient-directed information, whereas scores of 1 to 3 reflected varying levels of disagreement or uncertainty regarding the suitability of the response for patient education. In cases of rating discrepancy between evaluators, responses were reviewed jointly, and a consensus score was assigned. Inter-rater agreement between the two evaluators was assessed using Cohen’s kappa coefficient based on the initial independent ratings prior to consensus.

Descriptive statistics were used to summarize the distribution of expert agreement ratings. To examine whether differences existed in the distribution of reliability ratings among the AI platforms, a non-parametric Kruskal-Wallis H test was performed, given the ordinal nature of the Likert-scale data and the non-normal distribution of responses. The level of statistical significance was set at p < 0.05. All statistical analyses were conducted using SPSS Statistics version 29 (IBM Corp., Armonk, NY, USA).

## Results

A total of 30 individual evaluations were completed for each AI platform, corresponding to 15 patient-style questions independently assessed by two expert evaluators. Each evaluator reviewed all responses separately, resulting in 30 Likert-scale ratings per platform across the three AI chatbots evaluated (ChatGPT, Grok, and DeepSeek) (Table 2). Inter-rater reliability analysis demonstrated a Cohen’s kappa value of 0.85, indicating almost perfect agreement between the two evaluators. Overall, the distribution of expert agreement scores demonstrated a high level of concordance across all platforms. The majority of responses were rated as 5 (strongly agree), indicating strong expert agreement with the content and clarity of the AI-generated answers.

**Table 2:** 30 Likert-scale ratings per AI chatbot platform 1 = Strongly disagree, 2 =Disagree, 3 = Neutral, 4 = Agree, and 5 = Strongly agree

Investigator	Platform	Q no.1	Q no.2	Q no.3	Q no.4	Q no.5	Q no.6	Q no.7	Q no.8	Q no.9	Q no.10	Q no.11	Q no.12	Q no.13	Q no.14	Q no.15
First investigator	ChatGPT	5	5	5	5	5	5	4	5	5	5	5	5	5	5	5
	Grok	5	5	4	5	4	5	1	4	5	5	5	4	4	5	5
	DeepSeek	4	5	5	5	4	5	4	5	5	5	5	1	4	5	5
Second investigator	Grok	5	5	5	5	5	5	5	5	5	5	5	5	5	5	5
	ChatGPT	4	5	5	5	5	5	5	5	5	5	5	5	5	5	4
	DeepSeek	4	4	5	5	5	5	5	5	5	5	5	5	5	5	5

For ChatGPT, 27 responses were rated as 5 (strongly agree), while the remaining three responses received a score of 4 (agree). No responses generated by ChatGPT were assigned scores of 1, 2, or 3. Grok demonstrated similarly high agreement, with 24 responses receiving a score of 5 and five responses rated as 4. One response was assigned a score of 1, reflecting a single instance of low expert agreement. DeepSeek showed comparable performance, with 23 responses rated as 5 and six responses rated as 4. One response received a score of 1, while no responses were assigned scores of 2 or 3.

To assess whether differences existed in the distribution of expert agreement scores among the three AI platforms, a Kruskal-Wallis H test was performed. The analysis revealed no statistically significant differences in agreement distributions across ChatGPT, Grok, and DeepSeek (H = 2.05, df = 2, p = 0.36). These findings indicate that the three AI chatbots demonstrated comparable levels of expert-rated agreement when responding to patient-style questions related to endodontic apical lesions.

## Discussion

The present study evaluated the reliability and clinical appropriateness of responses generated by publicly accessible AI-based chatbot platforms when addressing patient-oriented questions related to endodontic apical lesions. As patients increasingly rely on digital tools to seek health-related information, understanding the quality and suitability of AI-generated responses has become clinically relevant, particularly in fields such as endodontics, where radiographic findings often require professional interpretation.

Previous research has primarily focused on the application of AI in supporting healthcare professionals, including its role in diagnosis, treatment planning, outcome prediction, and dental education [6-8]. However, the expanding use of AI tools by patients themselves should not be overlooked, as these platforms are now widely accessible and frequently consulted for medical and dental concerns. The effectiveness of AI-generated information in this context depends not only on its factual reliability but also on the patient’s ability to accurately describe symptoms and understand the information provided. Consequently, evaluating AI chatbot responses from a patient-education perspective represents an important and timely area of investigation [9,10].

The responses generated by all three AI chatbots demonstrated generally high levels of expert-rated reliability when evaluated using patient-oriented questions. ChatGPT achieved the highest proportion of favorable ratings, whereas Grok and DeepSeek exhibited comparable performance, albeit with a tendency toward simpler and more accessible language. These findings should be interpreted in light of the study design, as the evaluations were conducted by two dental specialists who assessed the suitability of responses from a patient-education perspective rather than diagnostic accuracy.

Previous investigations have reported variable performance among different AI models depending on the clinical context and evaluation framework. Recent studies have shown that ChatGPT and DeepSeek demonstrate strong performance in professional knowledge retrieval and primary case analysis when compared with Grok [11]. Similarly, ChatGPT showed the most stable and consistent functioning in dental implantology-related question answering and outperformed human dentists in implantology certification [12,13].

Yilmaz et al. reported that ChatGPT o1 achieved the highest performance among different ChatGPT versions when evaluated using oral pathology-related questions, whereas DeepSeek demonstrated stronger performance in knowledge-based assessments [14]. In contrast, another recent study found that the DeepSeek model achieved higher overall scores compared with ChatGPT-4o, while also acknowledging that both platforms retain potential for further improvement [15].

Although these findings appear heterogeneous, it is important to note that both studies evaluated AI performance primarily within the context of dental education and examination preparation. This differs fundamentally from the present study, which focuses on patient-oriented communication and the suitability of AI-generated responses for lay understanding rather than academic or examination-based performance.

Many international institutes and organizations, including the National Institutes of Health (NIH), Centers for Disease Control and Prevention (CDC), National Governors Association (NGA), and others, have promoted and encouraged using simple language to help patients to understand health information more easily and get them involved in their health-related decisions. It has been recommended that health materials should be clear, brief, and organized to ensure patient comprehension [16-18]. Based on expert evaluation, Grok and DeepSeek responses were perceived to use simpler and more accessible language compared with ChatGPT. On the other hand, Grok clarifies answers to the patients in clear and organized bullet points, which we consider a better way to be understood. Another benefit of Grok and sometimes DeepSeek is providing suggestions to the patient to go to a specialized doctor. This would keep involving the doctor in the process of patient education and decision-making of treatment.

Variation was observed in expert agreement ratings across individual questions when responses from the three AI platforms were considered collectively. Questions associated with the highest agreement scores were compared with those receiving lower scores according to their thematic category and question type. Notably, no consistent pattern emerged to explain differences in ratings, suggesting that response quality was not systematically influenced by the nature of the question or its classification.

Shaari et al. found that ChatGPT produced a higher level of information than recommended, which results in a lack of comprehension by regular patients [19]. Another study revealed that the natural language processing results of ChatGPT were basically consistent and suitable. However, areas of enhancement are still present [20]. Mago and Sharma concluded that ChatGPT, particularly ChatGPT-3, can be a brilliant utility for the community in raising understanding of different diseases and reducing the anxiety of the patients [21]. However, one of the critical problems related to AI use in healthcare is the diminished empathetic relationship delivered by the doctor-patient relationship, an important part of a successful therapeutic process. This significant value can't be obtained from AI utilization [22].

Although the majority of responses received high agreement scores, a small number of responses were rated lower by expert evaluators. Qualitative review of these responses revealed that lower ratings were primarily associated with incomplete or less precise clinical explanations, particularly when distinguishing between odontogenic and non-odontogenic causes. In some instances, responses lacked sufficient clarity regarding appropriate next steps or did not strongly emphasize the importance of professional dental evaluation. Additionally, certain responses were considered overly general or insufficiently tailored to the clinical context presented in the question. Importantly, no responses were identified as containing clearly harmful, misleading, or unsafe clinical advice. These findings suggest that while AI chatbots provide generally reliable patient-oriented information, limitations remain in delivering consistently precise and clinically nuanced guidance.

The primary limitation of this study is that AI-generated responses were evaluated by dental specialists, whereas the intended audience of these tools is the general patient population. Although expert assessment is valuable for judging clinical appropriateness, dentists’ familiarity with endodontic concepts may have influenced their perception of response clarity and suitability for lay users. Consequently, the findings primarily reflect professional evaluation rather than direct patient comprehension. Additionally, each question was submitted only once per platform. Given the non-deterministic nature of LLMs, repeated submissions of identical prompts may yield variable responses. Therefore, the findings reflect a single-instance evaluation and may not capture the full range of possible outputs generated by each system.

Future studies incorporating assessments by individuals without medical or dental training would provide a more representative evaluation of patient understanding. Additionally, study designs that include both clinicians and patients as evaluators may help delineate differences between professional judgments of reliability and patient perceptions of clarity and usefulness.

## Conclusions

This study demonstrates that publicly accessible AI chatbots can provide generally reliable and clinically appropriate responses to patient-oriented questions related to endodontic apical lesions. ChatGPT generated more comprehensive informational content, whereas Grok and DeepSeek tended to employ simpler, more patient-friendly language. These findings support the potential role of AI chatbots as adjunct tools for patient education, while reinforcing that clinical interpretation and treatment decisions should remain the responsibility of dental professionals.
